# Comparison of the Thermal Behavior and Chemical Composition of Milk Powders of Animal and Plant Origin

**DOI:** 10.3390/foods14030389

**Published:** 2025-01-24

**Authors:** Thomas Dippong, Laura Elena Muresan, Lacrimioara Senila

**Affiliations:** 1 Department of Chemistry and Biology, Faculty of Science, Technical University of Cluj-Napoca, 76A Victoriei St., 430122 Baia Mare, Romania; dippong.thomas@yahoo.ro; 2 Raluca Ripan’ Institute for Research in Chemistry, Babes Bolyai University, Fantanele, 30, 400294 Cluj-Napoca, Romania; laura_muresan2003@yahoo.com; 3 Research Institute for Analytical Instrumentation Subsidiary, National Institute of Research and Development for Optoelectronics Bucharest INOE 2000, 67 Donath Street, 400293 Cluj-Napoca, Romania

**Keywords:** milk powder, TGA analysis, volatiles, fatty acids, sugars

## Abstract

The present study aims to perform a comparative analysis of the chemical composition and thermal behavior of two distinct milk types, namely animal and plant-based. The thermal analysis revealed the presence of the following classes of compounds: hydrocarbons, heterocycles, aldehydes, ketones, amines and alcohols. All types of milk contain saturated fatty acids (SFAs), monounsaturated fatty acids (MUFAs) and polyunsaturated fatty acids (PUFAs), though the relative proportions of these vary depending on the specific milk type. Animal milk powders contain SFAs, including palmitic, stearic, and myristic acids, as well as moderate amounts of MUFAs, such as oleic and palmitoleic acids. They also contain lower PUFAs, including linoleic and alpha-linolenic acids. In contrast, plant-based milk powders, particularly soy milk powder, are rich in both linoleic and alpha-linolenic acids. Plant-based milk typically exhibits lower levels of SFAs and higher levels of MUFAs and PUFAs when compared to milk of animal origin. In conclusion, the fatty acid profiles of animal and plant-based milk powders reflect the different nutritional attributes and health implications associated with each. Thermal behavior analysis offers insights into the stability and potential flavor changes that may occur during processing and storage. The comparative analysis highlights significant differences in the chemical composition and thermal behavior of animal and plant-based milk powders.

## 1. Introduction

Milk powder is an excellent source of energy, rich in macro- and micronutrients. The nutritional value of powdered milk can be enhanced by adding functional bioactive compounds such as probiotics, prebiotics, human milk oligosaccharides, vitamins, minerals, taurine, inositol, osteopontin, lactoferrin, gangliosides, carnitine, etc. [1]. The composition of powdered milk consists of carbohydrates, lipids, proteins, vitamins, and minerals [1]. Milk under powdered form requires reconstitution with water before consumption. The preparation of powdered milk involves balancing macronutrients (proteins, fats, and lactose) and micronutrients (vitamins and minerals) [2].

During the process, water vapors from lactose are volatilized, leading to lactose crystallization and the formation of saturated lactose solution bridges between adjacent particles [3]. When lactose crystallization occurs, the milk molecules are rearranged, and hydrogen bonds are disrupted, resulting in the phase separation of the milk components, and forming cracks on the particle surfaces. From these cracks, fatty acids migrate, enriching the surface lipid layer. Since lipids are rich in fatty acids, they melt during heating [3]. Upon cooling, the fatty acids undergo complete or partial crystallization, forming solid lipid bridges [3].

The animal milk powder, obtained from cow, mare, donkey, goat or camel, remains an essential component of many food products and is widely used in the food industry [4]. Animal milk powder contains milk protein fatty acids, vegetable protein, carbohydrates, and vitamins [4,5]. Camel milk powder form contains water, proteins, casein and whey proteins, lactose, fats, minerals and vitamins, phospholipids, cholesterol, and triglycerides [6]. Lipids in camel milk powder present lower levels of saturated fatty acids, which are beneficial in reducing the risk of heart disease, as well as higher levels of monosaturated fatty acids and polyunsaturated fatty acids [7]. Furthermore, the high palmitic acid content of camel milk powder is beneficial for digestion and triglyceride absorption in infants [8]. The content of mono- and polyunsaturated fatty acids is higher in camel milk when compared to bovine milk [9]. Camel milk contains saturated, monounsaturated, and polyunsaturated fatty acids. Goat milk powder exhibits lower levels of casein and higher levels of fatty acids than cow milk powder, thereby providing nutritional support for infants. Goat milk powder contains a high concentration of caproic, caprylic, and capric acids, which impart an animal-like flavor [10]. Mare milk is comparable to breast milk in composition, with low concentrations of protein and fat and high concentrations of lactose, then that observed in cow, goat and sheep milk powder assortments [11]. The following minerals have been identified in mare milk: potassium, sodium and magnesium and α-linolenic acid. The content of casein, lactalbumin and serum albumin is 62%, which is significantly lower than the same proteins in buffalo, sheep, goat or donkey milk powder [12]. The low allergenic level of donkey milk is attributed to its protein fraction [12]. The digestibility of β-lactoglobulin in donkey milk powder is higher than that of buffalo milk powder, due to the high whey protein content and low casein content [13,14]. About 70% of cow milk contains saturated fatty acids [4]. In goat and cow milk powders, the proteins responsible for stabilizing fat micelles are whey proteins and casein, which result in thermal coagulation stability and emulsification stability during milk powder processing [4]. The content of saturated fatty acids is higher in the composition of fat globules of bovine and caprine milk, which is about 60% higher than in those of breast milk. However, the content of unsaturated fatty acids in the composition of fat globules of human milk is seven times higher [4]. Fat globules in donkey milk are very small (1.92 μm), and because of their small size they are distributed over a larger surface area, which is necessary for the action of lipase, resulting in higher digestibility [4].

The plant-based milk alternative market represents the fastest-growing segment in the global development of new functional and specialty beverage food products. Factors such as cow milk allergies, lactose intolerance, concerns about caloric intake, the prevalence of hypercholesterolemia, and an increasing preference for vegan diets have significantly influenced consumer choices, driving the demand for plant-based alternatives. These alternatives are also economically accessible options for lower-income groups in developing countries and regions with limited access to animal-derived milk. The consumption of plant-based products growth rate of 12.9% [15].

Plant-based milks are broadly classified into five categories based on the current market offerings: nut-based milks, cereal-based milks, seed-based milks, pseudocereal-based milks, and legume-based milks [16].

Vegetable powders, derived from plants such as soy, coconut, alfalfa, buckwheat or oats, have become increasingly popular due to their nutritional benefits and compatibility with various dietary preferences, including vegetarian and vegan diets [1,2]. Soy-based milk powder can ensure normal growth and is considered an acceptable alternative to cow milk powder [16]. Soy-based milk powder accounts for approximately 20–25% of infant feeding. Soy milk is prepared from soybeans and is a rich source of protein, vitamins, and minerals, including vitamin B12, calcium, magnesium, phosphorus, potassium, iron, and zinc. Oat milk, on the other hand, is prepared from oats and water and is fortified with nutrients, including calcium, vitamins, and potassium [5,17,18]. Oat milk contain a considerable quantity of fatty acids, proteins, minerals, vitamins, dietary fibers, and a multitude of micronutrients, which collectively confer several health benefits (the reduction of blood sugar and cholesterol levels, and the risk of cancer) [19]. Buckwheat is a rich source of starch, minerals, quercetin, phytosterols, lysine, glutamic acid, proline, and aspartic acid, which are used in the treatment of chronic diseases (e.g., diabetes, hypercholesterolemia, hypertension) [19,20]. Alfalfa is rich in amino acids, minerals, vitamins, dietary fiber, and β-carotene [21,22]. The oat milk powder is rich in carbohydrates, protein, fats, vitamins and minerals and fibers [23]. Coconut-based milk powder is a functional food because it provides lauric acid, which can help boost immunity against bacterial diseases, provides a stable emulsion when reconstituted with water, contains total fat, protein, calcium, and phosphorus [2,24].

Animal milk powders contain milk protein and animal fat, while vegetable powders contain vegetable protein, vegetable fat and carbohydrates [25]. From a nutritional point of view, animal milk powders are rich in calcium and vitamins, whereas vegetable powders may provide a wider range of nutrients such as plant protein and fiber [26].

The presence of unpleasant flavors can have a negative impact on the consumer experience and the quality of powdered milk, leading to product rejection [19]. Volatile compounds, which are responsible for the specific flavors and odors of these products, have become a topic of interest in recent research, as they can significantly influence the perception of taste and the quality of food products [27]. The flavor components of milk powder undergo numerous complex changes during processing. For instance, spray drying results in the release of free fat, which, through slight oxidation, imparts a sweet taste to the fatty and creamy flavor of milk powder. The most common classes of odorant volatile substances used in milk powders are aldehydes, ketones, alcohols, phenols, acids, heterocyclic compounds, and lactones [19]. The odorants responsible for non-enzymatic browning in powdered milk are 2-furaldehyde, 2-furfuryl butyrate and N-ethyl-2-formylpyrrole [19].

The primary source of energy in formula milk is lipids, providing almost half of an infant’s energy needs [4]. Milk fat is rich in bioactive fatty acids and plays an important role in consumption. There are also minimum requirements for essential fatty acids such as alpha-linolenic acid and linoleic acid, which infants cannot synthesize [4]. The most common fatty acids are the odd-chain fatty acids butyrate and conjugated linoleic acid [4]. Arachidonic acid and docosahexaenoic acid play a role in the development of plasma membrane components. In human milk, palmitic acid accounts for about 10% of the total energy intake of infants and is a key nutrient for the development of infant formula [4]. The recommended total lipid content for infant formula is 4.6–6.0 g/100 kcal, corresponding to about 40–54% of the energy content, which is similar to that found in human milk [28]. There are still marked differences in the triglyceride (TAG) and fatty acid (FA) composition between various brands of infant formula based on ruminant skim milk supplemented with coconut, maize, soy, palm, sunflower, peanut, and rapeseed oils [29]. In addition to providing energy, triglycerides play an important role in milk flavor [14]. The composition and content of fatty acids and triglycerides in milk depends mainly on the species, the environment, and the lactation period [29]. Romeu-Nadal studied the shelf life of infant formulas by measuring hydroperoxides, volatile compounds, fatty acid content, and sensory quality, concluding that storage temperature and time, as well as the polyunsaturated fatty acid content, are the most important factors in determining oxidation stability [30]. There are more than 400 fatty acids in milk fat. The health benefits of omega-3 have been studied extensively [9]. The composition of fatty acids differs based on the animal’s diet and on other environmental conditions. Due to its fatty acid content, camel milk has benefits such as anti-inflammatory and immunomodulatory effects. Oligosaccharides can prevent toxins and pathogenic bacteria from binding to target epithelial cells by exhibiting antibacterial activity [31].

Thermal analysis is based on the study of the physical and chemical changes undergone by chemical substances in solid food systems (e.g., powdered milk, coffee, cocoa, tea, and spices) and the changes that occur in these systems as a function of temperature variation. This analysis enables the identification and determination of their composition [32]. Thermal analysis can be used for the evaluation of the solid phases undergoing transformation as well as the gaseous phases that form [32]. Temperature affects the state, equilibrium, and kinetics of solid food systems by influencing the physical and chemical constants of substances [32]. The thermodynamic states of substances are determined by heat transfer mechanisms and termokinetic processes that cause variations in the physical and chemical properties of the substance. Heat treatments at elevated temperatures are commonly associated with drying processes and decomposition reactions, which invariably involve reversible or irreversible equilibria. Upon heating in inert atmospheres, an amplification of molecular motions occurs in solids [32]. If the intramolecular forces are weak, the substance decomposes, forming new molecular fragments, some or all of which are volatilized at the attained temperature [32]. The thermal analysis of milk powder conducted in food industries aimed to measure the changes in its properties as a function of temperature. The thermal analysis involves subjecting the milk powder samples to a controlled temperature program [33]. Malec [34] studied the loss of available lysine during the storage of milk powder or the prolonged heating of model systems derived from the composition of infant formulas. Schmitz described the lysine losses that occurred during the spray drying process, as well as the reaction kinetics for the high concentration regime at specific temperature–time conditions for spray drying [35]. The study of the thermal behavior of different milk powders showed that exothermic reactions correspond to the following processes: crystallization of amorphous lactose, Maillard reaction between milk proteins and lactose, oxidation of milk fat, and decomposition of lactose.

This study represents the first comprehensive comparison between animal- and plant-based milk varieties in terms of their chemical composition. The investigation includes a detailed analysis of fatty acids, volatile compounds, reduced sugars, and carbohydrates. Additionally, the thermal behavior of both milk types was evaluated and correlated with their chemical profiles to provide further confirmation of their compositional characteristics. The research also aims to classify and analyze the aroma profiles and volatile compounds based on their chemical classes and aroma types. The advantage of this study is that it provides a significant contribution to the field of food science and nutrition by conducting a detailed analysis of plant-based milk powder as a potential alternative to animal milk powder. This analysis is particularly relevant in the context of product development, particularly for infant, dairy allergy, lactose-free, cholesterol-free, and flavor-innovation products. Furthermore, a systematic classification and statistical analysis of fatty acid content and volatile compounds were performed. The findings of this study will contribute to a deeper understanding of the nutritional value of milk powders by analyzing their chemical composition and identifying differences in their nutrient profiles to understand the potential health benefits and to meet dietary needs. Additionally, the stability of these milk powders will be evaluated through thermal properties and behavior at elevated temperatures under various conditions. This research also highlights the potential applications of both plant-based and animal-derived milk powders in diverse food formulations, including their use in nutritional supplements, flavor enhancement, nutrient fortification, and as ingredients to improve the nutritional quality of flour-based or fortified food products.

## 2. Materials and Methods

### 2.1. Chemicals

The chemicals, including methanol, chloroform, sodium sulfate (Na_2_SO_4_), potassium chloride (KCl), isooctane, sodium hydrogen sulfate monohydrate (NaHSO_4_·H_2_O), ethanol, sodium chlorite (NaClO_2_), sodium hydroxide (NaOH), sulfuric acid (H_2_SO_4_), were purchased from Merck (Darmstadt, Germany). The Standard FAME Mixture (Supelco 37 Component FAME Mix, CRM47885) was purchased from Sigma-Aldrich (St. Louis, MO, USA) and used as a reference for identifying and quantifying fatty acid methyl esters in the samples. Ultrapure water was obtained from a Direct-Q3 UV Water Purification System (Millipore, Molsheim, France), used to ensure high-purity water for all the analyses.

### 2.2. Sample Description

Ten types of milk powder (500–1000 g) were purchased from specialized markets in Baia-Mare (Romania). The sample codes are as follows: ABC (camel, from UAE), ABCO (cow, from Romania), ABD (donkey, from Italy), ABG (goat, from Switzerland), ABM (mare, from France), PBO (oat, from Germany), PBS (soy, from Romania), PBA (alfalfa, from Romania), PBB (buckwheat, from the Czech Republic), PBC (coconut, from The Netherlands). Samples were stored in a dark, dry, and well-ventilated locker. All samples were freeze-dried (FreeZone 2.5 LiterBenchtop freeze dry system, Labconco, Kansas, MO, USA) at −40 °C and −25 psi for 24 h to uniformize their moisture content and then transferred to −20 °C and stored until further analysis.

### 2.3. Thermal Analysis

A quantity of 100 mg of milk powders from 10 different samples was measured and introduced in a special crucible in the derivatograph SDTQ600 (TA Instruments, New Castle, DE, USA). The samples included ABC (camel), ABCO (cow), ABD (donkey), ABG (goat), ABM (mare), PBO (oat), PBS (soy), PBA (alfalfa), PBB (buckwheat), and PBC (coconut). The curves of thermogravimetry (TG) and differential thermal analysis (DTA) were recorded. The registration of these curves was carried out by a SDT Q600 (TA Instruments, New Castle, DE, USA) instrument in air up to 1000 °C with a speed of 10 °C/min heating rate using alumina standards.

### 2.4. Lipid Extraction from Milk Powder and Gas Chromatography Analysis

Lipid milk powder samples were extracted using the method described in Senila et al. (2024), with adjustments [36]. Lipids were extracted using a mixture of methanol and chloroform, followed by phase separation with potassium chloride solution. The chloroform layer containing lipids was dried over sodium sulfate. Lipids were saponified using potassium hydroxide and then methylated using methanol and sulfuric acid to form FAMEs.

The FAME standard mixture was procured from Sigma-Aldrich. The FAMEs content was determined by gas chromatography-flame ionization detection (GC-FID; Agilent Technologies, 6890N, Santa Clara, CA, USA) with a ZB-WAX capillary column (30 m × 0.25 mm × 0.25 µm) and a flame ionization detector (FID; Agilent Technologies 7683, Santa Clara, CA, USA). The carrier gas was helium, with a constant flow rate of 1 mL min^−1^. The injection volume was 1 µL in a 1:20 split mode. The GC oven temperature program followed three stages. The temperature was maintained at 60 °C for one minute and then increased at a rate of 10 °C per minute until it reached 200 °C. Thereafter, it was increased at a rate of 5 °C per minute until it reached 220 °C. The injector and detector temperatures were set to 250 °C. The identification of FAs in samples was achieved by comparing their retention times with those of the Supelco FAME standard mixture.

### 2.5. Lipids Nutritional Quality Index

Quality indices were calculated based on the FAMES content according to Dongmo et al. [37]. The following indices were analyzed: atherogenic index (AI), thrombogenic index (TI), hypocholesterolemia/hypercholesterolemic ratio (h/H), health-promoting index (HPI), nutritive value index (NVI), polyene index (PI), n-3/n-6 PUFA, MUFA/SFA, and PUFA/SFA. The formulas used for quality indices calculation (AI, TI, h/H, HPI, NVI, and PI) are presented in Equations (1)–(6).

(1) $$AI=\frac{C12:0+\left(4\cdot C14:0\right)+C16:0}{\sum MUFA+\sum PUFA}$$

(2) $$TI=\frac{\left(C14:0+C16:0+C18:0\right)}{\left[(0.5\cdot \sum UFA)+(0.5\cdot \sum PUFA(n-6))+(3\cdot \sum PUFA\left(n-6\right))+\frac{\sum PUFA(n-3)}{\sum PUFA(n-6)}\right]}$$

(3) $$h/H=\frac{cisC18:1+\sum MUFA+\sum PUFA}{C12:0+C14:0+C16:0}$$

(4) $$HPI=\frac{\sum \left(MUFA+PUFA\right)}{\left[C12:0+\left(4\cdot C14:0+C16:0\right)\right]}$$

(5) $$NVI=\frac{C18:0+C18:1}{C16:1}$$

(6) $$PI=\frac{\sum PUFA}{\sum SFA}$$

### 2.6. Volatiles Analysis from Milk Powder Varieties by GC-MS

Volatile analysis of milk powder using gas chromatography-mass spectrometry (GC-MS) involves the extraction, identification, and quantification of volatile compounds that contribute to the flavor and aroma profiles of milk powders. Two grams of milk powder were introduced into a 20 mL headspace vial, and 10 mL of distilled water were added to rehydrate the milk powder. Two grams of NaCl were added to the vial to facilitate the extraction of volatile compounds. The GC oven temperature was set at 35 °C (held for 1 min), increased to 100 °C (held for 1 min) at 5 °C/min rate, and then 150 °C (held for 3 min) at 7 °C/min, and finally increased to 250 °C (held for 1 min) with 10 °C/min rate. The transfer line temperature was set at 280 °C and the ion source temperature was set to 250 °C.

### 2.7. Determination of Carbohydrates from Milk Powder

Ultra-high performance liquid chromatography (UHPLC) (Agilent Technologies, Santa Clara, CA, USA) was used to analyze the sugars from milk powder hydrolysates. The content of sugars was analyzed by UHPLC (1260 Infinity II, Agilent Technologies, Santa Clara, CA, USA), which contains a quaternary pump (Agilent Technologies, G7111B, 1260 Infinity II, Santa Clara, CA, USA), an Agilent Autosampler with an injection valve fitted with a 20 µL sample loop. The separation was performed on a 5 µm Polaris NH_2_ 250 µm × 4.6 mm (Agilent Technologies, Santa Clara, CA, USA). The column temperature (Agilent Technologies 1290 Infinity II Multicolumn Thermostat, Santa Clara, CA, USA) was kept constant at 30 °C, and the mobile phase flow rate was 0.6 mL min^−1^. The Evaporative Light Scattering Detector (ELSD) (Agilent Technologies, 1290 Infinity ELSD, Santa Clara, CA, USA) has the following characteristics: nebulization temperature of 70 °C, evaporation temperature of 90 °C, and gas flow of 1.2 SLM. The eluent used was acetonitrile–water (75:25) with a flow rate of 0.6 mL/min and an injection volume of 20 µL. All the samples were filtered through a 0.45 µM PTFE filter for LC analysis.

### 2.8. Determination of Reduced Sugars in Milk Powder

The milk powder samples were hydrolyzed with 1% H_2_SO_4_ for reducing sugar analysis. A total of 10 mg of samples were mixed with 50 mL of 1% H_2_SO_4_ and heated at 100 °C for 2 h. The hydrolysates obtained were filtered, and the pH of the solutions was neutralized to 7 with 5 M NaOH. The reduced sugars were determined by the colorimetric method by using a DNS reagent (3,5-dinitrosalicylic acid) [38].

### 2.9. Statistical Analysis

Data were statistically analyzed using Origin Software (version 2020b, OriginLab, Northampton, MA, USA). Each analysis was performed in triplicate, and standard deviations were calculated. A Tukey test was conducted following the ANOVA to perform pairwise comparisons among groups. The different letters indicate a statistically significant difference at a level of *p* ˂ 0.05. A multivariate statistical method, namely hierarchical cluster analysis, was used to ascertain the correlation between variables and group similarity. Pearson’s correlation coefficient was performed to measure the linear relationship between variables, and principal component analysis was performed to ascertain the correlative effects of principal components.

## 3. Results and Discussion

### 3.1. Thermal Analysis of Milk Powder Varieties

**Figure 1:**
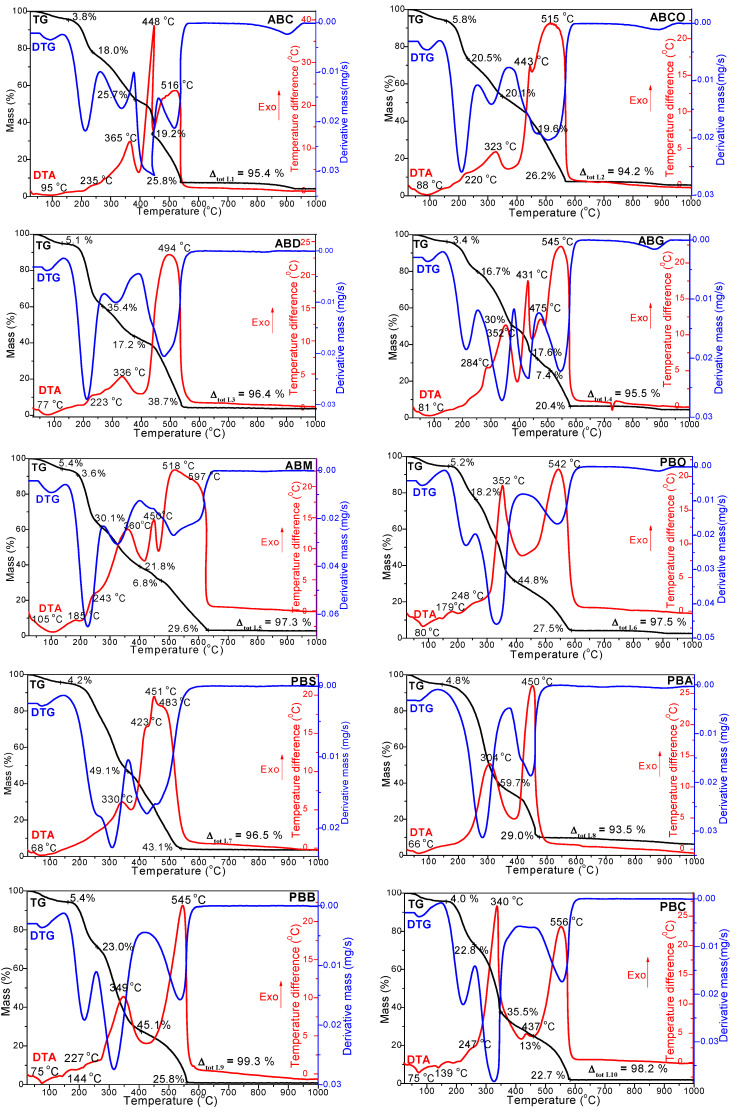
TG, DTG, and DTA curves for ABC (camel), ABCO (cow), ABD (donkey), ABG (goat), ABM (mare), PBO (oat), PBS (soy), PBA (alfalfa), PBB (buckwheat), PBC (coconut) in milk powder varieties.

Figure 1 shows the thermal decomposition up to 1000 °C of milk powders from animal and vegetable sources.

Camel (ABC) and cow (ABCO) powdered milk presents five distinct thermal effects. (i) the endothermic effect observed at temperatures between 88 and 95 °C is attributed to the drying of powder formula and the desorption of physically absorbed water molecules (at these temperature values, the heating of the shell can be related to moisture and water linked with the structure evaporation phenomenon [39]), as evidenced by the TG curve, exhibited a mass loss of 3.8–5.8%; (ii) an exothermic effect was observed at temperatures between 220 and 235 °C, accompanied by a mass loss between 18.0 and 20.0% that can be attributed to the decomposition of volatile organic compounds (VOCs); (iii) the exothermic effect was observed between 323 and 365 °C and is attributed to the decomposition of saturated and monounsaturated fatty acids, resulting in a mass loss of 20.5–25.7%; (iv) the exothermic effect was observed between 443 and 448 °C, accompanied by a mass loss of 19.2 to 19.6%, and is attributed to the decomposition of polyunsaturated fatty acids and proteins; (v) the exothermic effect was observed between 515 and 516 °C and can be attributed to the decomposition of carbohydrates, which resulted in a mass loss of 25.8 to 26.2% [31,39,40,41]. The low thermal stability of cow milk powder (ABCO) may be due to increased ionic calcium and low pH, resulting in low thermal stability and the formation of an undesirable solid coagulum during sterilization [4].

The thermal curves of donkey milk powder (ABD) show four effects: (i) the endothermic effect at 77 °C due to the drying and the desorption of physically absorbed water, with a mass loss of 5.1%; (ii) the exothermic effect observed at 223 °C, accompanied by a mass loss of 35.4%, that can be attributed to the decomposition of VOC and sugars; (iii) the exothermic effect observed at 336 °C, which can be attributed to lipid decomposition, with a mass loss of 17.2%; (iv) the large exothermic effect observed at 494 °C with a mass loss of 38.7% that can be attributed to protein and carbohydrate breakdown [31,40,41,42,43].

Goat milk powder (ABG) exhibits six thermal decomposition effects: (i) the endothermic effect at 81 °C, with mass loss of 3.4%, is due to the loss of water; (ii) the exothermic effect at 284 °C, with mass loss of 16%, is attributed to the decomposition of volatile substances and reducing sugars; (iii) the exothermic effect at 352 °C is attributed to the decomposition of saturated and monounsaturated fatty acids, with mass loss of 30%; (iv) the sharp exothermic effect at 431 °C with a mass loss of 17.6% is attributed to the decomposition of proteins and polyunsaturated fatty acids; (v) the exothermic effect at 475 °C is due to the decomposition of starch, having a mass loss of 7.4%; and (vi) the broad exothermic effect at 545 °C with a mass loss of 20.4% is attributed to the decomposition of dextrose and sucrose [31,40,41,42,43].

In mare milk powder (ABM), there are seven thermal decomposition effects observed (7): (i) the endothermic effect at 105 °C with a mass loss of 5.4%, attributed to the drying of milk powder; (ii) the exothermic effect at 185 °C with a mass loss of 3.6% is due to the decomposition of sugars; (iii) the exothermic effect at 243 °C with a mass loss of 30.1% is due to the decomposition of volatile compounds and sugars; (iv) the exothermic effect at 360 °C is attributed to the decomposition of lipids with a mass loss of 21.8%; (v) the exothermic effect at 450 °C with a mass loss of 6.8% is attributed to the decomposition of polyunsaturated fatty acids and proteins; and (vi–vii) the exothermic effects at 518 and 597 °C are due to the decomposition of carbohydrates (dextrose, sucrose, and starch), having a mass loss of 29.6% [31,40,41,42,43].

In addition to the endothermic effect of structural water loss at 75–80 °C, oat (PBO) and buckwheat (PBB) powdered milks also exhibit four exothermic effects: at 144–179 °C, with a mass loss of 5.2–5.4% (attributed to sugar decomposition); at 227–248 °C, with a mass loss of 16.7–23.0% (attributed to the decomposition of volatile compounds); at 349–352 °C, with a mass loss of 44.8–45.1% (attributed to the decomposition of fatty acids); and at 542–545 °C, with a mass loss of 25.8–27.5% (attributed to the decomposition of proteins and carbohydrates).

Soymilk powder (PBS) exhibits five thermal decomposition effects: (i) the endothermic effect observed at 68 °C, with a mass loss of 4.2%, due to water loss; (ii) the exothermic effect at 330 °C, with a mass loss of 49.1%, is attributed to the decomposition of volatile substances, reducing sugars and fatty acids (lipids); (iii–v) the exothermic peaks at 423, 451, and 483 °C, with a mass loss of 43.1%, which are attributed for the first time to the separate decomposition of the three major components of the carbohydrate class: starch, sucrose, and dextrose, as well as the decomposition of proteins [31,40,41,42,43]. Alfalfa powdered flakes (PBA) show the least thermal effects. Besides the thermal effect of drying the powder formula and the desorption of physically absorbed water molecules (66 °C, 4.8%), only two exothermic effects are observed: at 304 °C (with a mass loss of 59.7%), attributed to the decomposition of sugars, volatiles, and lipids, respectively, and at 450 °C (with a mass loss of 29%), attributed to the decomposition of proteins and carbohydrates [31,39,40,41,42,43]. Coconut milk powder (PBC) presents six distinct thermal effects of decomposition: (i) The endothermic peak at 75 °C is attributed to the loss of water consistency; (ii) The exothermic peak at 139 °C is due to the decomposition of sugars. This additional stage may be due to the high lactose content, indicating the possible course of the Maillard reaction in the mixture with proteins simultaneously, leading to the loss of crystallization; (iv) The exothermic peak at 340 °C is attributed to the decomposition of saturated and monosaturated fatty acids, which results in a mass loss of 33.5%; (v) The sharp exothermic peak at 437 °C, with a mass loss of 13.0%, is attributed to the decomposition of polyunsaturated fatty acids and proteins; (vi) The sharp exothermic peak at 556 °C, with a mass loss of 22.7%, is attributed to the decomposition of carbohydrates. The total mass loss ranges from 93.5% to 99.3%, in the following order PBA < ABCO < ABC < ABG < ABD <PBS < ABM < PBO < PBC < PBB, the varieties being consistent with their composition.

The activation energy necessary for the thermal decomposition of a mixture of components depends on the energy barrier for the individual constituents [44]. In the case of milk powder, the activation energy for thermal decomposition is influenced by the chemical composition of each milk variety [44]. In the course of the first stages, the activation energy required for the lactose degradation was almost similar for the different samples of milk [44]. According to Sunooj et al. [44], data concerning the thermal decomposition of cow and camel milk showed that the degradation of fat and protein occurred in almost the same temperature range and, thus, the activation energy needed for thermal degradation could not be determined separately.

In our previous studies on coffee [41,42,43], the thermal analysis revealed various transformations in coffee composition, namely, drying, water loss and decomposition of lipids, polysaccharides, amino acids, and proteins. Thermal analysis also revealed transformations in cocoa powder’s composition [41,42,43]: drying and water loss; decomposition of polysaccharides, proteins, and lipids; and crystalline phase transformations and carbonizations.

### 3.2. HS-SPME GC-MS Analysis of Volatile Organic Compounds

The volatile compounds of five animal and five vegetable milk powders were analyzed by SPME followed by GC-MS, as shown in Table 1. A total of 27 volatile aromatic compounds were identified, divided into five classes of volatile compounds: 10 hydrocarbons (33.3%), seven heterocycles (24.1%), four aldehydes (21.5%), two ketones (11.5%), two amides (6.0%), and two alcohols (3.7%), as shown in Table 2. Hydrocarbons had the highest proportion (33.3%, Table 2) in the samples analyzed and induced sweet, pungent, citrus, gasoline, fruity, camphorous, or woody odors [45,46,47]. 2,4-Hexadiene, which is more abundant in vegetable-based milk powders, with the highest concentration in alfalfa-based milk powder (PBA, 23.2%, Table 1 and Table 2), imparts a strong sweet green flavor [45]. Cycloheptene, which is present only in mare milk powder (ABM, 17.7%), has a citric, slightly pungent aroma like the odor of unsaturated hydrocarbons [34]. α-pinene, present only in buckwheat milk powder (PBB, 12.3%), imparts a lemon-sweet, fresh, camphor, sweet, terpene aroma to PBB milk powder [47]. 1-octene, present only in arrowroot milk powder (ABD, 20.9%), imparts a green, olefinic aroma [29]. Cyclohexene is found only in lucerne-based milk powder (PBA, 3.5%) and has a mild, sweet, woody, and almond flavor [47]. Toluene is only present in buttermilk milk powder (ABD, 2.3%) and gives this type of milk powder a sweet flavor [47].

The presence of heterocyclic compounds (24.1%, Table 2) in the milk powders analyzed imparts sweet, astringent, nutty, balsamic, and floral flavors to the milk powders. Methylpyrazine, present only in camel milk powder (ABCO, 4.3%), has a strong green and toasted nutty flavor with a sweetish aftertaste [45]. Azetidine is found in the highest concentrations in several types of milk powders, with the highest concentration in goat milk powder (ABG, 52.2%); it has an astringent ammonia taste [38]. Izoquinoline, present in several types of milk powders, with the highest concentration in oat-based milk powders (PBO, 9.5%), is a liquid, almost colorless, slightly viscous compound with a sweet, balsamic–herbal and slightly floral aroma [45]. Pyrrole is present in small amounts; the highest concentration is found in mare milk powder (ABM, 1.3%) and gives a sweet, slightly astringent, burning, propyl-formate-like, sweet, slightly astringent aroma [48].

Aldehydes are derived from the oxidative degradation of unsaturated fatty amino acids or the hydrolysis of triglycerides and are readily oxidized in contact with air, hence the rancid off-flavor in milk powder samples [19,45,49]. The specific aldehyde flavors are green, herbal, sweet, astringent, woody, fruity, and citrus. Hexanal is an active flavor compound, an important indicator of lipid oxidation, found in high concentrations in soy-based milk powders (PBS, 57.5%). It has a very strong, penetrating aroma with aromatic nuances of sweet, green, herbal, fatty, grassy, fruity, woody vegetable, apple, and citrus [19]. In extreme dilutions, hexanal is more reminiscent of freshly cut grass and unripe fruit, especially apple and plum, and its sour notes at high concentrations resemble the rancid taste of butter [45]. Higher concentrations of propanal are found in lucerne-based milk powder (PBA, 14.7%). It is a colorless liquid with a strong sweet, ethereal and vinegary aroma, and it is used in the synthesis of intermediates in the chemical and pharmaceutical industries and in the production of flavors and fragrances [19,45,49]. Crotonaldehyde is found in low concentrations (ABM, 0.5%) and is used as a preservative and flavoring agent due to its sweet astringent flavor [50]. 2,4-Hexadienal is derived from the auto-oxidation of polyunsaturated fatty acids of plant origin. It is present in the highest concentration in lucerne-based milk powder (PBB, 23.2%) and imparts a green, sweet flavor [51].

Ketones are the distinctive flavor components of milk powder (11.5%, Table 2), contributing to green, sweet, fruity, and floral flavors when present in sufficient amounts. They appear by β-oxidation or thermal degradation of unsaturated fatty acids, post-oxidative decarboxylation of free fatty acids, or by the degradation of amino acids. Ketones impart a pleasant flavor to milk [19,52]. 2-Pentanone is found in the highest concentration in mare milk powder (ABM, 40.6%) and has a pleasant, diffuse green flavor, milder than that of acetone [45]. 4-Hexanolide is a lactone, most abundant in coconut-based milk powder (PBC, 60.4%). It imparts a sweet, herbal flavor [46].

The content of amides (6.0%) in milk powders is associated with an astringent taste, both for acrylamide (the highest content is found in camel milk powder (ABC), 30.3) and acetamide (it is found in the highest concentration (5.1%) in oat-based milk powder, PBO) [46]. Alcohols (3.7%, Table 2) are significant contributors to the aroma profile, imparting notes of sweetness, green, fruitiness, and citrus. 1-Octanol, present only in donkey milk powder (ABD, 7.8%), evinces green, fruity, citrus, floral, fresh, orange-rose sweet, herbal, waxy orange-rose mushroom flavors [52]. Additionally, the second alcohol, butoxyethanol (28.6%), which imparts a sweet flavor, is only present in donkey milk powder.

Table 2 illustrates that milk powder samples derived from animal milk have the highest proportion of volatile hydrocarbon compounds (24.9%), whereas plant-based milk powder samples present the highest proportion of aldehydes (33.8%). Amide content is markedly higher in animal milk powder samples (9.1%) than in vegetable milk powder samples (2.9%). If heterocyclic compounds are preserved in the two major types of milk powders, the ketone content is only present in the milk powder samples obtained from animal sources (8.9%).

Table 3 illustrates the flavor profiles of the 10 milk powder varieties, which were classified into four categories: pungent (32.6%), green (32.3%), sweet (22.0%), and gasoline (13.1%). The flavor profile of plant products, due to their inherent character, imprints a strong green flavor (43.6) onto the milk powder products. In contrast, the green flavor content is significantly lower (21.0%) in milk powder samples derived from animal sources. In the case of animal milk powder samples, the pungent flavor is the most prevalent (37.0%), while in plant-based milk powder samples, the pungent flavor accounts for 28.1% of the total flavor profile. The sweet flavor is significantly more pronounced in milk powder samples of vegetable origin (28.3%) than in those of animal origin (15.7%). The gasoline flavor is exclusively observed in milk powder samples of animal origin (26.3%), which is likely attributed to the higher lipid content.

Table 4 illustrates the percentage analysis of the flavor profiles for the samples used in this study. Each segment indicates the proportion of volatile compounds with each flavor analyzed individually for all milk powder assortments. The distribution of sweet flavor in milk powder assortments shows the decrease in sweet flavor content of coconut milk powder (29.61%) in the following order: PBC > ABD > PBB > PBA > PBS > PBO > ABM > ABG > ABC > ABCO. Coconut milk powder (PBC) has the highest contribution to sweetness at 29.61%, indicating a high content of sweet volatile compounds. This characteristic makes it ideal for use in desserts or products requiring a pronounced sweet profile. Donkey (ABD) and buckwheat (PBB) milk powders contribute 16.86% and 13.60%, respectively, and are also suitable for products with a sweet taste. Cow milk powder (ABCO) and goat milk powder (ABG) have much lower contributions of 0.14% and 4.41%, respectively, indicating a reduced presence of sweet volatile compounds in these varieties. These differences are important for the development of specific food products where the flavor profile is crucial.

The green flavor, generally associated with freshness and vegetal notes, is dominated by cow milk powder (ABM 24.25% concentration) and soymilk powder (PBS 21.78% concentration). The green flavor content decreases in percentage in the following order: ABM > PBS > PBB > PBO > PBA > ABD > ABG > ABCO > ABC > PBC. These green volatile compounds are essential for products that need to reproduce a fresh, vegetal taste. Buckwheat milk powder (PBB, 18.43%) and oat milk powder (PBO, 14.50%) make significant contributions due to their specific flavor. Lesser contributors to the green flavor profile are camel milk powder (ABC) and coconut milk powder (PBC), with 1.32% and 0.84%, respectively, thus reflecting the low presence of these flavors and making them less suitable for applications where a green flavor profile is desired.

The astringent flavor is strongly present in camel (ABC) and goat (ABG) milk powders and decreases in the following order: ABG > ABC > PBO > PBA > PBC > LPBB > PBS > ABD > ABCO > ABM. This flavor profile is often associated with harsher tastes that may be desirable in certain foods or beverages. The significant contributions of oat (PBO) and alfalfa (PBA) milk powders, 12.99% and 11.58%, respectively, indicate the presence of volatile compounds that give them flavor. In contrast, cow milk powder (ABM) and donkey milk powder (ABD) contribute with very low percentages of 0.69% and 2.48%, making them less suitable for products requiring an astringent, pungent profile.

Cow and donkey milk powders are characterized by a significantly higher lipid content compared to the other varieties analyzed. This nutritional characteristic directly contributes to the improvement of the creamy taste perception due to the ability of lipids to improve texture and enhance flavor profiles. According to Table 4, cow milk powder (ABCO) clearly dominates this category with a contribution of 66.09%, followed by donkey milk powder (ABD) with 33.91%. These significant values are indicative of a high lipid concentration, which favors a pronounced creaminess sensation, essential in the formulation of food products aiming at a rich texture and a pleasant taste experience.

Based on the analysis above, we can summarize that coconut and donkey milk powders dominate the sweet flavor category, making them ideal for products requiring a sweet taste; cow and soy milk powders are essential to give products a fresh, vegetal note; for astringent flavors, camel and goat milk powders are best; for creaminess, cow and donkey milk powders are superior due to their high lipid content, providing a rich texture and flavor that is essential in many food applications. This information is useful for developing and optimizing food products according to the desired flavor profile.

### 3.3. GC-FID Analysis of FAMEs Content

The content of lipids varied in the following order (%): 23.6 (PBB) > 20.45 (PBO) > 15.3 (PBC) > 7.7 (PBS) > 4.98 (ABM) > 4.14 (ABC) > 3.8 (ABG) > 3.4 (ABCO) > 2.8 (PBA) > 0.8 (ABD). The highest lipid content was found in buckwheat (PBB) and oat (PBO) milk powders, making them the richest sources of lipids among the samples analyzed, while donkey milk powder (ABD) had the lowest lipid content.

The powder milk fat quantities and the fatty acid compositions identified in different plant-based and animal milk powder samples are presented in Table 5. SFAs, MUFAs, and PUFAs were identified in all samples. In camel milk (ABC), palmitic acid (C16:0) is the predominant SFA, accounting for 31.7% of the total, followed by stearic acid (C14:0), which constitutes 12.89% of the total, and myristic acid (C14:0), which represents 12.89% of the total. The results obtained are consistent with those previously reported by Leparmarai et al. [53], which indicated that fatty acid content was approximately 10% C14:0, 29.38% for C16:0, and 10.97% for C18:0. A total of 61.6% of the fatty acids in camel milk are SFAs, 30.4% are MUFAs, and 7% are PUFAs. The content of palmitic acid in camel milk is approximately three times higher than in cow, buckwheat, and coconut milk. Caproic acid (C6:0) was found in fats of goat and cow milk dairy products. It has an important role in metabolism due to metabolization by the body through the production of energy and antimicrobial properties. C13:0 fatty acids are relatively rare in nature compared to other saturated fatty acids but are found in dairy and vegetable oils. They are found in low levels in camel, coconut, goat, mare, and buckwheat milk powder. The FAs found in donkey milk (ABD) are: 21.2% C16:0 > 15.08% C18:1 > 10.47% C12:0 > 11.32% C10:0 >8.67% > 6.77% > C8:0C14:0 5.14% > C18:0 > 4.25% C18:2. Goat milk (ABG) is composed of 28.28% C16:0, 20.53% C18:1, 11.58% C14:1, 10.63% C10:0, 9.87% C8:0, and 5.34% C12:0. The results obtained in this study are consistent with those previously reported by Ediriweera [54]. The study reported that goat milk is a rich source of fatty acids, with palmitic acid (C16:0) as the predominant saturated fatty acid, followed by oleic acid (C18:1), linoleic acid (C18:2), and α-linolenic acid (C18:3n3). The fatty acid composition of mare milk (ABM) differs from that of cow milk. The SFAs from mare milk are palmitic acid (17.8%), myristic acid (10.42%), lauric acid (5.65%), capric acid (4.48%), and a small amount of stearic acid (2.40%). Oleic acid was found to be the predominant fatty acid with 24.45% and linoleic acid with 19.8%. A total of 6.41% was found to be α-linolenic acid. Cow milk (ABCO) contains a variety of SFA, MUFA, and PUFA fatty acids. The SFAs amount to 60.7%, with predominant C16:0 (15.92%), C18:0 (12.09%), C14:0 (9.74%), C12:0 (4.64%), C10:0 (4.34%), and C8:0 (3.79%). The content of SFAs identified in cow milk and reported by Alvarez-Hess et al. (2024) was 73.7% [55]. According to Hu et al. [56] and Alvarez-Hess et al. [55], palmitic and oleic acids are two important fatty acids in cow milk.

Soy milk (PBS) has the highest omega-3 content of all milk powders and has the following health benefits: brain health, heart improvement, reduced menopausal symptoms, as well as being a good source of vitamin A, vitamin B, potassium, calcium, retinol, folate, and choline. The main PUFAs found are C18:2(c+t)(n6) (58.37%), C18:1(c+t)(n9) (15.65%), C16:0 (11.65%), C18:3(n3) (7.39%), and small amounts of C18:0 (3.41%) and C14:0 (1.66%). Soy milk contains low levels of SFA and higher levels of PUFAs. Lucerne (PBA) is another name for alfalfa, a legume used as animal feed. The FAs found in alfalfa milk are C16:0 (40.38%), C18:0 (12.53%), C18:1(c+t)(n9) (8.72%), C14:0 (4.90%), and C18:2(c+t)(n6) (4.60%). The highest amount of SFAs was found in lucerne milk. The highest SFAs are found in buckwheat milk (PBB). The predominant FAs found in buckwheat milk are lauric acid (C12:0 −38.27%), myristic acid (C14:0 −14.72%) and palmitic acid (C14:0). Buckwheat milk is consumed for its properties such as maintaining skin health, supporting the immune system, and regulating inflammation. Coconut milk (PBC) is notable for its high concentration of saturated fatty acids. Among these, lauric (39.33%), myristic (14.96%), and palmitic acids (11.14%) are particularly present. Of the various saturated fatty acids present in coconut milk, lauric acid is particularly abundant and is known to possess a range of health benefits, including antimicrobial properties. It is important to note that coconut milk is a rich source of fats. However, it is essential to consume it in moderation as part of a balanced diet, as excessive intake of saturated fats may not be optimal for health. Nevertheless, the specific composition of fatty acids in coconut milk can vary depending on factors such as the processing method and the variety of the coconut used. It has been demonstrated that elevated levels of α-linoleic acid may contribute to an increased risk of lipid peroxidation and, consequently, a deterioration in the stability of the milk powder [28].

The highest concentration of lauric acid (C12:0) was found in ABM (oat), followed by ABCO (coconut) and PBB (buckwheat). According to Rindsig and Schultz [57], lauric acid is an antiviral and antibacterial agent because lauric acid is transformed into monolauril compounds. The highest concentration of SFAs was found in buckwheat (PBB) and oat (PBO). Vegetable milks, except for soy milk, have a much higher content of saturated fatty acids. Plant-based milks, such as soy, coconut, oat, alfalfa, and buckwheat, make a significant difference due to their high omega 6 and omega 3 content. Coconut milk (PBC) is not a good source of omega 3, but has a high content of MUFAs, which can have health benefits. Also, oats and buckwheat can be a dairy-free alternatives to milk products.

**Figure 2:**
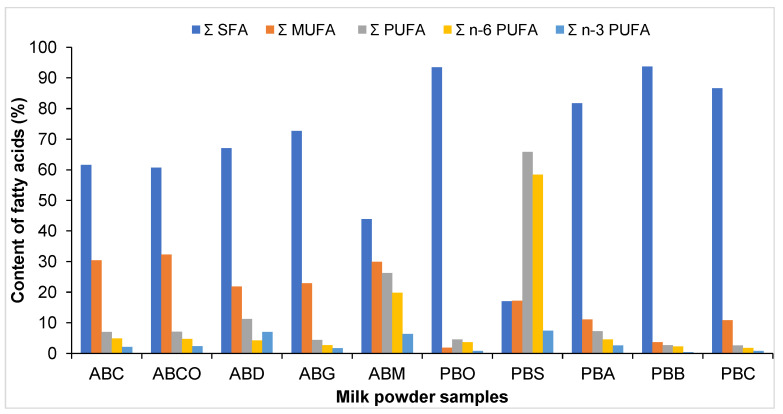
SFA, MUFA, and PUFA content of animal and vegetable milk powders.

Among animal milks, goat milk contains the highest proportion of saturated fatty acids (SFAs). The monounsaturated fatty acid (MUFA) content in animal milk powder varies (Figure 2) in the following order: cow (ABCO, 32.3%) > camel (ABC, 30.4%) > mare (ABM, 29.9%) > goat (ABG, 22.9%) > donkey (ABD, 21.8%). The highest content of PUFAs (Figure 2) was identified in mare milk (26.2%). The composition of fatty acids can vary between different animal milks. Camel (ABC) and mare (ABM) milk have the highest contents of MUFAs. Nevertheless, mare milk (ABM) is distinguished by its particularly high content of polyunsaturated fatty acids (PUFAs), which are also of great importance for human health. Regarding the differences between fatty acid compositions, the highest content of omega 3 was found in donkey (ABD, 7.0%) and mare milk (ABM, 6.4%). These findings imply that donkey and mare milk may offer supplementary health advantages in comparison to other dairy products due to their elevated omega-3 content.

Nutritional quality indices of milk varieties. Table 6 illustrates the calculated nutritional indices for milk powder varieties. The ratio of monounsaturated to saturated fatty acids (MUFA/SFA) ranged from 0.02 (PBO) to 1.01 (PBS). This value was used to evaluate the impact on cardiovascular health. A high PUFA/SFA ration was found in PBS and is considered beneficial for cardiovascular health as polyunsaturated fats are known to lower bad cholesterol levels and improve heart health, whereas high levels of saturated fats can increase the risk of heart disease. A high omega 3/omega 6 was identified in ABD, so consumption of donkey milk can offer a better balance that favors anti-inflammatory and cardioprotective effects. The atherogenic index (AI) was calculated based on lauric, myristic, and palmitic acids reported to the total amount of unsaturated fatty acids. The recommendation is to consume milk with an AI of 1 for optimal cardiovascular health. AI values significantly above 1 suggest higher atherogenic potential due to higher proportions of lauric, myristic, and palmitic acids.

Consumption of milk powder from oat (PBO), buckwheat (PBB), and coconut (PBC) is recommended with moderation due to the high SFA acid content. A lower TI is a principal indicator of the thrombogenic potential of fatty acids. In this study, all milk varieties, except for soy milk (PBS) and mare milk (ABM), exceed the value of 1. The ratio of hypocholesterolemia to hypercholesterolemic (h/H) represents a significant indicator used in the evaluation of the potential impact of fatty acids on cholesterol levels. It is recommended that this ratio be maintained at a level greater than 1. Health-promoting index (HPI) is another parameter that evaluates the balance of fatty acids in milk varieties. Like h/H indices, it is recommended to be higher (than 1). At such a level, it suggests a higher proportion of unsaturated fatty acids. Mare milk (ABM) and soy milk (PBS) have HPIs higher than 1 [58]. The ratio between the sum of stearic and oleic acid reported to palmitoleic acid gives the nutritive value index [58]. Goat, soy, and coconut milk have higher NVI, which is more beneficial for human health. The PI indicates the susceptibility of oil to peroxidation; a lower PI is desirable. All samples analyzed have low PI.

### 3.4. Reduced Sugars and Carbohydrates from Milk Powder

The reduced sugars and carbohydrates were quantified, and the highest reduced sugar concentration was identified in oat milk powder (PBO), at 18.0%. This was followed by alfalfa (13.2%), goat (10.0%), camel (8.0%), cow (5.3%), soy (4.5%), donkey (4.1%), and coconut (2.3%). The carbohydrate content of the milk powders was found to be as follows: 60.2% (PBB), 35.2% (PBS), 30.2% (PBA), 23.0% (PBC), 21.0% (ABM), 13.0% (ABG), 6.2% (ABD), 3.0% (ABC), and 2.3% (ABCO). The lipid and carbohydrate content of buckwheat and oat milk powders make them well-suited to provide high-energy nutrients for individuals with high energy requirements. Camel milk powder (ABC) and cow milk powder (ABCO) present the lowest carbohydrate content, 3.0% and 2.3%, respectively. Each type of milk powder offers distinctive nutritional advantages, rendering them suitable for various health and dietary necessities. Insoluble dietary fiber is a complex polysaccharide system that includes cellulose, hemicellulose, and lignin. These constituents serve as primary building blocks of the cell wall microfibrils. The presence of insoluble dietary fiber in milk powder can enhance nutritional value and offers various health benefits, such as digestive health, increased satiety, blood sugar control, and cancer prevention [59]. Carbohydrates are one of the main macronutrients in soymilk powder. Soy milk (PBS) powder contains carbohydrates, mainly in the form of sugars and fiber. The exact amount of carbohydrates can vary depending on factors such as brand, processing methods, and any added ingredients. Soybeans are a good source of fiber, and this is retained in soymilk powder. The fiber in soymilk powder contributes to digestive health and can help regulate blood sugar and cholesterol levels. The carbohydrate content of cow milk (ABCO) is approximately 50–55% and contains preponderate lactose, 40–45% for goat milk and 65–70% for donkey milk powder.

Donkey milk has a higher lactose content than cow and goat milk, which contributes to its higher carbohydrate content [59]. According to the literature, carbohydrates in various types of milk powders and beverages, including camel milk powder, coconut milk powder, oat milk powder, soymilk powder, alfalfa milk powder, and buckwheat milk powder, are as follows: 35–40% in camel, 20–30% in coconut, 60–70% in oat, 30–35% in soy, 20–25% in alfalfa, and 50–60% in buckwheat [11]. Tulashie et al. (2022) studied the production of coconut milk as alternative-based milk and reported the following results: 135.95 kcal, 14.12 g total fat content, 0.70 g total sugars, 0.15 g reducing sugars, and 0.55 g of sucrose [60].

### 3.5. Principal Component Analysis

**Figure 3:**
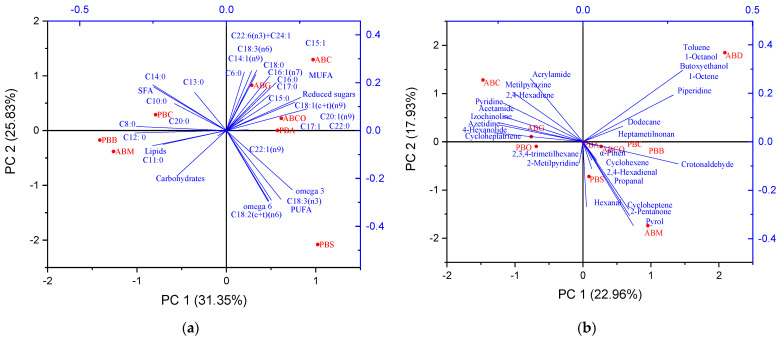
Principal component analysis loading plot of PC1 and PC2 for fatty acids (**a**) and volatiles (**b**) of different milk powder varieties.

The PCA analysis of the milk powder samples provides valuable insights into the variation and grouping based on their fatty acid profiles and other nutritional components. Principal Component 1 (PC1) accounts for 31.35% of the total variance, while Principal Component 2 (PC2) accounts for 25.83% (Figure 3a). The PCA plot demonstrated that samples ABC, ABCO, ABG, and PBA are clustered and exhibit elevated levels of MUFA and specific SFAs, including C18:0, C17:0, C15:0, C16:0, and C22:0, as well as a distinctive PUFA, C22:6(n3). The samples (ABC, ABCO, ABG, and PBA) are beneficial due to their high MUFA content, which is associated with improved cardiovascular health. The presence of DHA (C22:6(n3)) further enhances their nutritional profile, providing essential omega-3 fatty acids. The ABM and PBB are grouped based on high levels of reduced sugars, carbohydrates, lipids, and some specific SFAs (C12:0 and C11:0). The presence of medium-chain SFAs (C12:0, C11:0) is beneficial for quick energy metabolism but should be balanced with other dietary fats to avoid excessive intake of SFAs. PBS are distinguished by a notable presence of PUFAs (C18:2(n6), C18:3(n3), C22:1(n9)). PBS are rich in essential PUFAs, including omega-6 (C18:2(n6)) and omega-3 (C18:3(n3)), which are important for reducing inflammation and supporting heart health. Erucic acid (C22:1(n9)), though beneficial in small amounts, should be monitored due to potential health concerns at high levels. The sample (PBC) was characterized by some SFA (C14:0, C13:0, C8:0, C20:0, and C10:0).

PC scores represent the similarity or variance between milk powder varieties based on their volatile profiles.

About volatile content, samples ABC and ABG exhibit similarities with respect to acrylamide, 2,4-hexadiene, acetamide, 4-hexanolide, izochinoline, azetidine, and cycloheptatriene. This group comprises acrylamide, aromatics, and nitrogen content, which exert a significant influence on the overall flavor and aroma of the samples, imparting distinctive characteristics. The second group, comprising samples ABCO, ABM, PBS, PBA, and PBB, displays similarities in the content of pinene, 2,4-hexadienal, cyclohexadiene, crotonaldehyde, cyclohexane, propanal, cycloheptene, hexanal, 2-pentanone, and pyrol (Figure 3b). Additionally, ABD exhibits a distinctive profile regarding the composition of alcohols, aromatic hydrocarbons, ethers, and aliphatic hydrocarbons (1-octanol, toluene, 1-octene, butoxyethanol, piperidine, dodecane, heptametilnonan, and 2,3,4-trimetilhexane).

**Figure 4:**
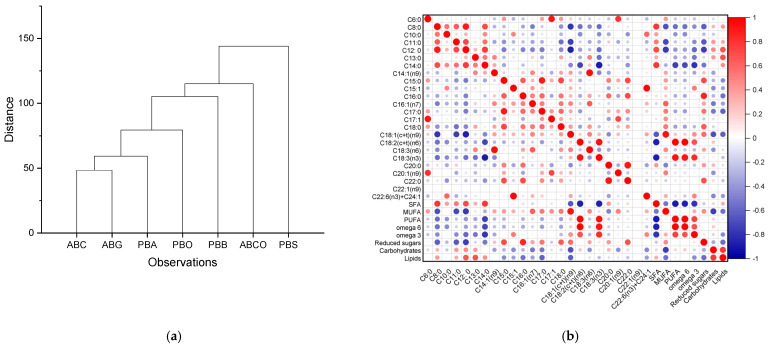
(**a**) Dendrogram of hierarchical clustering of milk powder samples based on their fatty acids composition (**b**) and Pearson correlation.

The dendrogram was constructed for hierarchical clustering using links based on Euclidean distance and is presented in Figure 4a. The following clustering was observed: one group (camel and goat), PBA, PBO, PBB, ABCO, and PBS are linked to each other. The soy sample (PBS) was found to be different from all other samples in terms of fatty acid content, especially C18:2 (n6) (omega-6 fatty acids) and C18:3 (n3) (omega-3 fatty acids).

## 4. Conclusions

The study compared the chemical composition and thermal behavior of animal and plant-based milk powders. Thermal decomposition differed significantly, with plant-based powders showing distinct carbohydrate decomposition patterns and the highest total mass loss in buckwheat milk powder (99.3%). Animal milk powders contained higher saturated fatty acids (SFAs), while plant-based powders, particularly soymilk powder, had elevated levels of monounsaturated and polyunsaturated fatty acids (MUFAs and PUFAs), which are associated with health benefits. Flavor profiles also varied, with plant-based powders being sweeter and greener, while animal-based powders were more astringent and creamier. Both types of milk have unique advantages and can complement each other in a balanced diet. The volatiles present in the samples were found to encompass the following classes of compounds: hydrocarbons (33.3%), heterocycles (24.1%), aldehydes (21.5%), ketones (11.5%), amines (6.0%), and alcohols (3.7%), which contribute to the stability and flavor profiles. A comparative analysis of the two main categories of milk powders, animal milk powder and vegetable milk powder, reveals notable differences in the percentage distribution of chemical compounds across the various classes. In animal milk powder, the predominant chemical compounds are hydrocarbons (41.7%), heterocycles (23.8%), and aldehydes (9.2%), with amides representing a concentration like that of aldehydes (9.1%). In vegetable milk, the highest value is represented by aldehydes (33.8%), followed by hydrocarbons (24.9%), heterocycles (24.4%), and amides, which are present in a very low percentage compared to that in animal milk powder, representing only 2.9%. Both animal-based and plant-based milks have their own set of advantages and disadvantages. Animal-based milk contains essential fatty acids, whereas plant-based milk has a lower proportion of saturated fatty acids. Plant-based milk can adequately fulfill the nutritional requirements of humans, although a balanced approach may include both types of milk.

## Figures and Tables

**Table 1:** Volatile organic compounds, chemical formula, chemical group, odor type, and content (%) identified using HS-SPME GC-MS for ABC (camel), ABCO (cow), ABD (donkey), ABG (goat), ABM (mare), PBO (oat), PBS (soy), PBA (alfalfa), PBB (buckwheat), PBC (coconut). The data are expressed as averages ± standard deviation (*n* = 3).

VOC	Group	Odor	ABC	ABCO	ABD	ABG	ABM	PBO	PBS	PBA	PBB	PBC
Acetamide	amide	pungent	4.3 ± 0.3 ^b^	0.2 ± 0.01 ^ef^	0.1 ± 0.01 ^f^	<0.1	0.1 ± 0.01 ^f^	5.1 ± 0.4 ^a^	0.8 ± 0.07 ^de^	1.7 ± 0.1 ^c^	<0.1	1.2 ± 0.1 ^cd^
Acrylamide	amide	pungent	30.3 ± 2.5 ^a^	0.7 ± 0.08 ^c^	6.6 ± 0.5 ^b^	2.7 ± 0.2 ^c^	0.3 ± 0.03 ^c^	0.7 ± 0.06 ^c^	2.6 ± 0.21 ^c^	2.3 ± 0.2 ^c^	<0.1	<0.1
Azetidine	heterocyclic	pungent	31.2 ± 2.1 ^b^	0.5 ± 0.04 ^e^	1.4 ± 0.2 ^e^	52.2 ± 5.1 ^a^	0.4 ± 0.04 ^e^	20.5 ± 2.1 ^c^	10.1 ± 1.1 ^d^	24.5 ± 1.8 ^c^	3.6 ± 0.3 ^de^	6.8 ± 0.4 ^de^
Butoxyethanol	alcohol	sweet	<0.1	<0.1	28.6 ± 2.3 ^a^	<0.1	<0.1	<0.1	<0.1	<0.1	<0.1	<0.1
Crotonaldehyde	aldehyde	sweet	<0.1	<0.1	0.3 ± 0.03 ^b^	<0.1	0.5 ± 0.04 ^a^	<0.1	<0.1	<0.1	<0.1	<0.1
Cycloheptene	hydrocarbon	sweet	<0.1	<0.1	<0.1	<0.1	17.7 ± 1.5 ^a^	<0.1	<0.1	<0.1	<0.1	<0.1
Cycloheptatriene	hydrocarbon	pungent	9.3 ± 0.8 ^c^	5.6 ± 0.5 ^d^	<0.1	29.0 ± 2.5 ^a^	1.5 ± 0.1 ^ef^	16.0 ± 1.8 ^b^	2.2 ± 0.2 ^def^	9.2 ± 0.8 ^c^	4.5 ± 0.3 ^de^	17.5 ± 1.2 ^b^
Cyclohexene	hydrocarbon	sweet	<0.1	<0.1	<0.1	<0.1	<0.1	<0.1	<0.1	3.5 ± 0.3 ^a^	<0.1	<0.1
1-Octanol	alcohol	green	<0.1	<0.1	7.8 ± 0.7 ^a^	<0.1	<0.1	<0.1	<0.1	<0.1	<0.1	<0.1
Dodecane	hydrocarbon	gasoline	<0.1	86.9 ± 5.6 ^a^	23.7 ± 2.0 ^b^	<0.1	<0.1	<0.1	<0.1	<0.1	<0.1	<0.1
Heptametilnonan	hydrocarbon	green	<0.1	5.7 ± 0.4 ^a^	<0.1	<0.1	<0.1	<0.1	<0.1	<0.1	<0.1	<0.1
2,4-Hexadienal	aldehyde	green	<0.1	<0.1	1.0 ± 0.01 ^bc^	<0.1	<0.1	<0.1	2.8 ± 0.2 ^b^	23.2 ± 2.1 ^a^	<0.1	<0.1
2,4-Hexadiene	hydrocarbon	sweet	4.6 ± 0.3 ^a^	<0.1	<0.1	<0.1	<0.1	<0.1	<0.1	<0.1	<0.1	<0.1
Hexanal	aldehyde	green	<0.1	<0.1	<0.1	6.4 ± 0.5 ^e^	37.6 ± 2.8 ^c^	46.8 ± 3.2 ^b^	57.5 ± 4.5 ^a^	15.2 ± 1.4 ^d^	0.6 ± 0.05 ^e^	2.7 ± 0.2 ^e^
4-Hexanolide	ketone	sweet	4.1 ± 0.21 ^b^	<0.1	<0.1	<0.1	<0.1	<0.1	<0.1	<0.1	<0.1	60.4 ± 5.2 ^a^
Izochinoline	heterocycle	sweet	3.3 ± 0.25 ^d^	<0.1	0.1 ± 0.01 ^e^	8.4 ± 0.7 ^b^	<0.1	9.5 ± 0.8 ^a^	<0.1	0.5 ± 0.04 ^e^	<0.1	4.7 ± 0.3 ^c^
Metilpyrazine	heterocycle	green	4.3 ± 0.24 ^a^	<0.1	<0.1	<0.1	<0.1	<0.1	<0.1	<0.1	<0.1	<0.1
2-Metilpyridine	heterocyclic	sweet	<0.1	<0.1	0.1 ± 0.01 ^c^	1.3 ± 0.1 ^c^	<0.1	0.7 ± 0.06 ^c^	6.3 ± 0.5 ^b^	<0.1	17.6 ± 1.3 ^a^	<0.1
1-Octene	hydrocarbon	gasoline	<0.1	<0.1	20.9 ± 1.8 ^a^	<0.1	<0.1	<0.1	<0.1	<0.1	<0.1	<0.1
2-Pentanone	ketone	green	<0.1	<0.1	<0.1	<0.1	40.6 ± 3.4 ^a^	<0.1	9.6 ± 0.8 ^b^	<0.1	<0.1	<0.1
α-Pinen	hydrocarbon	sweet	<0.1	<0.1	<0.1	<0.1	<0.1	<0.1	<0.1	<0.1	12.3 ± 1.0 ^a^	<0.1
Piperidine	heterocycle	sweet	<0.1	<0.1	5.7 ± 0.4 ^a^	<0.1	<0.1	<0.1	0.4 ± 0.04 ^c^	4.4 ± 0.3 ^b^	<0.1	<0.1
Propanal	aldehyde	sweet	<0.1	<0.1	<0.1	<0.1	<0.1	0.6 ± 0.05 ^c^	4.6 ± 0.31 ^b^	14.7 ± 1.2 ^a^	<0.1	<0.1
Pyridine	heterocycle	pungent	8.7 ± 0.7 ^a^	<0.1	<0.1	<0.1	<0.1	<0.1	2.1 ± 0.2 ^c^	<0.1	2.5 ± 0.2 ^c^	6.8 ± 0.4 ^b^
Pyrol	heterocycle	sweet	<0.1	0.2 ± 0.01 ^c^	<0.1	<0.1	1.3 ± 0.1 ^a^	<0.1	0.6 ± 0.05 ^b^	0.6 ± 0.04 ^b^	<0.1	<0.1
Toluene	hydrocarbon	sweet	<0.1	<0.1	2.3 ± 0.2 ^a^	<0.1	<0.1	<0.1	<0.1	<0.1	<0.1	<0.1
2,3,4-trimetilhexane	hydrocarbon	green	<0.1	<0.1	1.4 ± 0.1 ^b^	<0.1	0.1 ± 0.01 ^b^	<0.1	0.4 ± 0.03 ^b^	0.2 ± 0.01 ^b^	58.9 ± 4.2 ^a^	<0.1

<Below limit of quantification (LQ). Values are presented as means ± standard division (SD). Means in the same row with different superscripts are significantly different (*p* ≤ 0.05).

**Table 2:** Classification of volatile compounds identified in milk powders by chemical classes for all milk powder products.

Chemical Class	Animal Milk Powder (%)	Plant-Based Milk Powder (%)
Hydrocarbons	41.7	24.9
Heterocycles	23.8	24.4
Aldehydes	9.2	33.8
Ketones	8.9	14.0
Amides	9.1	2.9
Alcohols	7.3	-
Total	100	100

**Table 3:** Classification of volatile compounds identified in milk powders by flavor profile for all milk powder products.

Flavor Profile	Animal Milk Powder (%)	Plant-Based Milk Powder (%)
Pungent	37.0	28.1
Green	21.0	43.6
Sweet	15.7	28.3
Gasoline	26.3	-
Total	100	100

**Table 4:** Percentage distribution of sweet, green, astringent, and creamy flavor in milk powder varieties.

	Flavor	Sweet (%)	Green (%)	Astringent (%)	Creamy (%)
Milk					
ABC		5.43	1.32	25.74	-
ABCO		0.14	1.77	2.16	66.09
ABD		16.86	3.17	2.48	33.91
ABG		4.41	1.98	25.75	-
ABM		8.85	24.25	0.69	-
PBO		4.93	14.50	12.99	-
PBS		5.40	21.78	5.46	-
PBA		10.78	11.96	11.58	-
PBB		13.60	18.43	3.25	-
PBC		29.60	0.84	9.90	-
**Total**		100.00	100.00	100.00	100.00

Note: ABC (camel), ABCO (cow), ABD (donkey), ABG (goat), ABM (mare), PBO (oat), PBS (soy), PBA (alfalfa), PBB (buckwheat), and PBC (coconut).

**Table 5:** Fatty acid profiles from milk powder (data are expressed in % (*w*/*w*/)) expressed as averages ± standard deviation (*n* = 3).

Acid Type		ABC	ABCO	ABD	ABG	ABM	PBO	PBS	PBA	PBB	PBC
*caproic acid*	C6:0	nd	3.57 ± 0.25 ^a^	nd	0.98 ± 0.08 ^b^	nd	nd	nd	nd	1.29 ± 0.1 ^b^	nd
*caprylic acid*	C8:0	0.46 ± 0.03 ^e^	3.79 ± 0.25 ^c^	6.77 ± 0.51 ^b^	3.19 ± 0.24 ^cd^	1.64 ± 0.1 ^de^	9.15 ± 0.85 ^a^	0.12 ± 0.01 ^e^	3.79 ± 0.2 ^c^	8.20 ± 0.6 ^ab^	7.75 ± 0.6 ^ab^
*capric acid*	C10:0	0.48 ± 0.03 ^d^	4.34 ± 0.3 ^c^	11.32 ± 1.23 ^a^	10.63 ± 1.23 ^a^	4.48 ± 0.3 ^c^	6.54 ± 0.5 ^b^	nd	nd	5.79 ± 0.4 ^bc^	5.45 ± 0.5 ^bc^
*undecanoic acid*	C11:0	nd	nd	nd	nd	nd	0.75 ± 0.04 ^a^	nd	nd	0.45 ± 0.04 ^b^	nd
*lauric acid*	C12: 0	1.11 ± 0.09 ^c^	4.64 ± 0.25 ^bc^	10.47 ± 1.02 ^b^	5.34 ± 0.41 ^bc^	5.65 ± 0.42 ^bc^	40.3 ± 2.5 ^a^	nd	4.52 ± 0.3 ^bc^	38.27 ± 2.8 ^a^	39.3 ±2.8 ^a^
*tridecanoic acid*	C13:0	0.25 ± 0.01 ^b^	nd	nd	0.18 ± 0.01 ^c^	0.25 ± 0.01 ^b^	nd	nd	nd	0.37 ± 0.04	0.13 ± 0.01
*myristic acid*	C14:0	11.37 ± 1.2 ^c^	9.74 ± 0.8 ^c^	8.67 ± 0.75 ^c^	11.58 ± 1.25 ^bc^	10.42 ± 1.3 ^c^	15.49 ± 1.2 ^a^	1.66 ± 0.1 ^d^	4.90 ± 0.4 ^d^	14.72 ± 1.2 ^a^	14.96 ± 1.2 ^d^
*myristoleic acid*	C14:1(n9)	0.22 ± 0.02 ^b^	nd	nd	0.15 ± 0.01 ^b^	0.40 ± 0.03 ^a^	nd	nd	nd	nd	0.16 ± 0.01 ^b^
*pentadecanoic acid*	C15:0	1.46 ± 0.1 ^c^	3.42 ± 0.21 ^b^	1.32 ± 0.15 ^e^	1.29 ± 0.13 ^c^	0.64 ± 0.05 ^d^	nd	nd	4.22 ± 0.4 ^a^	0.59 ± 0.04 ^d^	0.29 ± 0.01 ^de^
*pentadesenoic acid*	C15:1	0.39 ± 0.02 ^b^	nd	1.16 ± 0.13 ^a^	0.11 ± 0.01 ^d^	0.24 ± 0.01 ^c^	nd	nd	nd	nd	0.14 ± 0.01 ^cd^
*palmitic acid*	C16:0	31.67 ± 2.5 ^b^	15.92 ± 1.2 ^cde^	21.2 ± 2.2 ^c^	28.28 ± 2.4 ^b^	17.80 ± 1.23 ^cde^	18.56 ± 1.6 ^cd^	11.7 ± 1.2 ^e^	40.38 ± 3.8 ^a^	13.48 ± 1.2 ^de^	11.14 ± 1.1 ^e^
*palmitoleic acid*	C16:1(n7)	9.80 ± 0.85 ^c^	2.51 ± 0.18 ^bc^	3.26 ± 0.28 ^d^	0.65 ± 0.05 ^b^	4.23 ± 0.3 ^d^	0.1 ± 0.01 ^d^	0.32 ± 0.03 ^c^	2.35 ± 0.18 ^d^	0.42 ± 0.05 ^d^	0.12 ± 0.01 ^d^
*heptadecanoic acid*	C17:0	1.93 ± 0.1 ^b^	2.94 ± 0.16 ^a^	2.08 ± 0.2 ^b^	0.84 ± 0.07 ^c^	0.45 ± 0.03 ^cde^	0.83 ± 0.07 ^c^	0.05 ± 0.002 ^e^	2.76 ± 0.15 ^a^	0.51 ± 0.04 ^cd^	0.14 ± 0.02 ^de^
*cis-10-hepta-decenoic acid*	C17:1	0.75 ± 0.06 ^d^	3.96 ± 0.2 ^a^	1.31 ± 0.15 ^c^	0.44 ± 0.03 ^de^	0.13 ± 0.01 ^ef^	nd	0.55 ± 0.04 ^de^	nd	2.13 ± 0.21 ^b^	0.15 ± 0.02 ^ef^
*stearic acid*	C18:0	12.89 ± 1.1 ^a^	12.09 ± 1.3 ^ab^	5.14 ± 0.42 ^ef^	9.87 ± 0.56 ^bc^	2.40 ± 0.17 ^gh^	nd	3.41 ± 0.21 ^fg^	12.53 ± 0.15 ^a^	9.16 ± 0.84 ^cd^	7.01 ± 0.6 ^de^
*oleic acid*	C18:1 (n9)	19.0 ± 1.2 ^bc^	17.69 ± 1.5 ^bc^	15.08 ± 1.3 ^c^	20.53 ± 1.8 ^ab^	24.45 ± 2.2 ^a^	1.89 ± 0.12 ^e^	15.7 ± 1.2 ^c^	8.72 ± 0.8 ^d^	1.13 ± 0.15 ^e^	6.80 ± 0.52 ^d^
*linoleic acid*	C18:2 (n6)	4.24 ± 0.3 ^c^	4.69 ± 0.3 ^c^	4.25 ± 0.32 ^c^	2.29 ± 0.16 ^c^	19.80 ± 1.5 ^b^	3.75 ± 0.2 ^c^	58.37 ± 4.2 ^a^	4.60 ± 0.42 ^c^	2.28 ± 0.26 ^c^	1.60 ± 0.02 ^c^
*γ-linolenic acid*	C18:3(n6)	0.68 ± 0.05 ^a^	nd	nd	0.44 ± 0.02 ^b^	nd	nd	nd	nd	nd	0.24 ± 0.02 ^c^
*α-linolenic acid*	C18:3(n3)	0.84 ± 0.04 ^d^	2.40 ± 0.16 ^c^	2.52 ± 0.15 ^c^	0.43 ± 0.03 ^ef^	6.41 ± 0.4 ^b^	0.82 ± 0.07 ^d^	7.39 ± 0.62 ^a^	2.65 ± 0.26 ^c^	0.38 ± 0.03 ^de^	0.15 ± 0.02 ^f^
*arachidic acid*	C20:0	nd	0.20 ± 0.01 ^e^	nd	0.38 ± 0.02 ^d^	0.16 ± 0.01 ^e^	1.94 ± 0.2 ^b^	0.12 ± 0.01 ^e^	4.48 ± 0.5 ^a^	0.82 ± 0.07 ^c^	0.36 ± 0.03 ^d^
*gondoic acid*	C20:1(n9)	0.24 ± 0.01 ^e^	8.12 ± 0.67 ^a^	1.02 ± 0.13 ^c^	1.01 ± 0.12 ^c^	0.24 ± 0.02 ^e^	nd	0.65 ± 0.05 ^d^	nd	nd	3.46 ± 0.21 ^b^
*behenic acid*	C22:0	nd	nd	nd	0.11 ± 0.01 ^b^	nd	nd	nd	4.10 ± 0.51 ^a^	nd	nd
*erucic acid*	C22:1(n9)	nd	nd	nd	nd	0.20 ± 0.02 ^a^	nd	nd	nd	nd	nd
*docosahexanoic + nervonic acid*	C22:6(n3) + C24:1	1.25 ± 0.1 ^b^	nd	4.44 ± 0.34 ^a^	1.27 ± 0.01 ^b^	nd	nd	nd	nd	nd	0.62 ± 0.05 ^c^

Note: ABC (camel), ABCO (cow), ABD (donkey), ABG (goat), ABM (mare), PBO (oat), PBS (soy), PBA (alfalfa), PBB (buckwheat), PBC (coconut). Data are expressed as mean ± standard deviation (*n* = 3). Values indicated with different letters were significantly different from each other at *p* ≤ 0.05 levels, whereas the same letters showed no significant differences (*p* > 0.05). Different letters in each column showed a significant difference at the level of *p* ≤ 0.05. nd—not determined.

**Table 6:** Nutritional quality indices of milk varieties.

Index	ABC	ABCO	ABD	ABG	ABM	PBO	PBS	PBA	PBB	PBC
MUFA/SFA	0.49	0.53	0.33	0.31	0.68	0.02	1.01	0.14	0.04	0.13
PUFA/SFA	0.11	0.12	0.17	0.06	0.60	0.05	3.87	0.09	0.03	0.03
$\frac{\sum PUFA(n-3)}{\sum PUFA(n-6)}$	0.42	0.51	1.64	0.62	0.32	0.22	0.13	0.58	0.17	0.42
AI	2.09	1.51	2.01	2.93	1.16	18.42	0.22	3.52	17.45	8.21
TI	1.70	1.14	1.28	2.30	0.36	2.37	0.08	2.60	3.74	2.70
h/H	0.59	0.82	0.65	0.55	1.50	0.09	6.12	0.32	0.06	0.14
HPI	0.85	1.30	0.82	0.60	1.66	0.09	6.23	0.37	0.10	0.21
NVI	3.3	11.9	6.2	46.8	6.3	18.9	59.6	9.0	24.5	115.1
PI	0.04	nd	0.21	0.04	nd	nd	nd	nd	nd	0.06

Note: ABC (camel), ABCO (cow), ABD (donkey), ABG (goat), ABM (mare), PBO (oat), PBS (soy), PBA (alfalfa), PBB (buckwheat), and PBC (coconut).

## Data Availability

The original contributions presented in this study are included in the article. Further inquiries can be directed to the corresponding author.
